# Moving neuromuscular disorders research forward: from novel models to clinical studies

**DOI:** 10.1242/dmm.044370

**Published:** 2020-02-25

**Authors:** Maaike van Putten, Julija Hmeljak, Annemieke Aartsma-Rus, James J. Dowling

**Affiliations:** 1 Department of Human Genetics, Leiden University Medical Center, Albinusdreef 2, 2333 ZA Leiden, The Netherlands; 2 Disease Models & Mechanisms, The Company of Biologists, Bidder Building, Station Road, Histon, Cambridge CB24 9LF, UK; 3 Program for Genetics and Genome Biology, The Hospital for Sick Children, Peter Gilgan Centre for Research and Learning (PGCRL), Bay St., 14th Floor, Toronto, ON M5G 0A4, Canada; 4 Departments of Paediatrics and Molecular Genetics, University of Toronto, Toronto, ON M5G 1X8, Canada

**Keywords:** Disease modelling, Muscular dystrophy, Neuromuscular disease, Rare disease, Translational research

## Abstract

Neuromuscular disorders (NMDs) encompass a diverse group of genetic diseases characterized by loss of muscle functionality. Despite extensive efforts to develop therapies, no curative treatment exists for any of the NMDs. For multiple disorders, however, therapeutic strategies are currently being tested in clinical settings, and the first successful treatments have now entered clinical practice (e.g. spinraza for spinal muscular atrophy). Successful clinical translation depends on the quality and translatability of preclinical findings and on the predictive value of the experimental models used in their initial development. This Special Issue of Disease Models & Mechanisms has a particular focus on translational research for NMDs. The collection includes original research focusing on advances in the development of novel *in vitro* and *in vivo* models, broader understanding of disease pathology and progression, and approaches to modify the disease course in these models. We also present a series of special articles and reviews that highlight our understanding of cellular mechanisms, biomarkers to tract disease pathology, the diversity of mouse models for NMDs, the importance of high-quality preclinical studies and data validation, and the pitfalls of successfully moving a potential therapeutic strategy to the clinic. In this Editorial, we summarize the highlights of these articles and place their findings in the broader context of the NMD research field.

## Abstract (precis)

**Summary:** This Editorial summarizes the highlights of DMM's Special Issue ‘A Guide to Using Neuromuscular Disease Models for Basic and Preclinical Studies’.


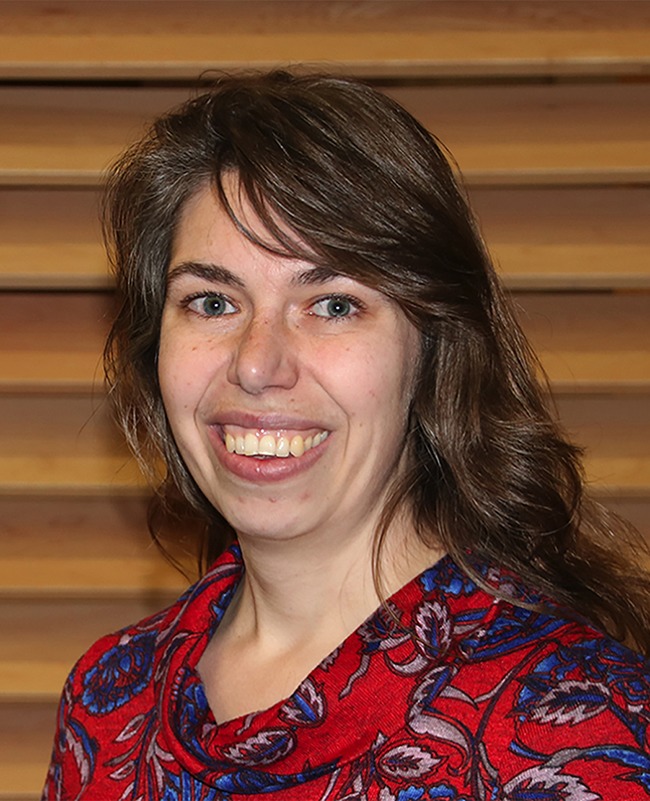


**Maaike van Putten**


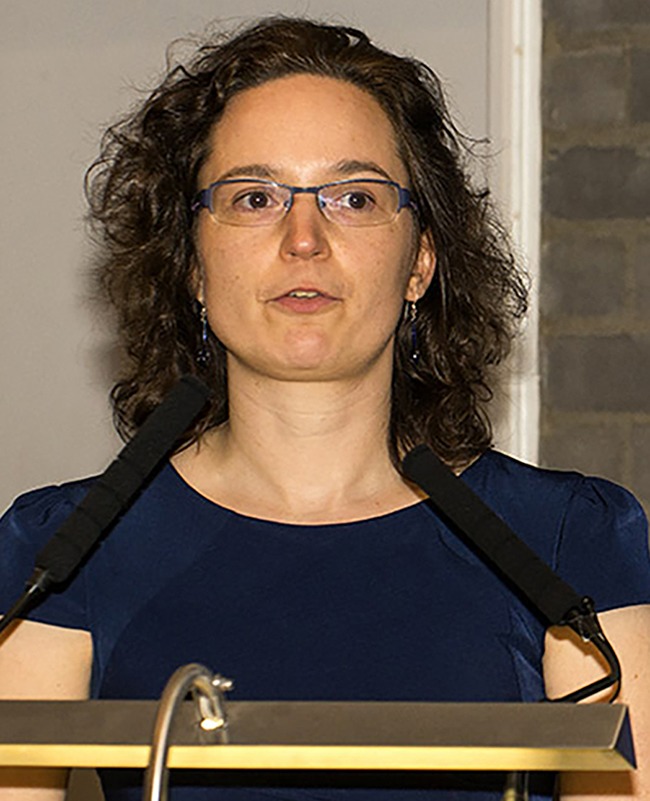


**Annemieke Aartsma-Rus**


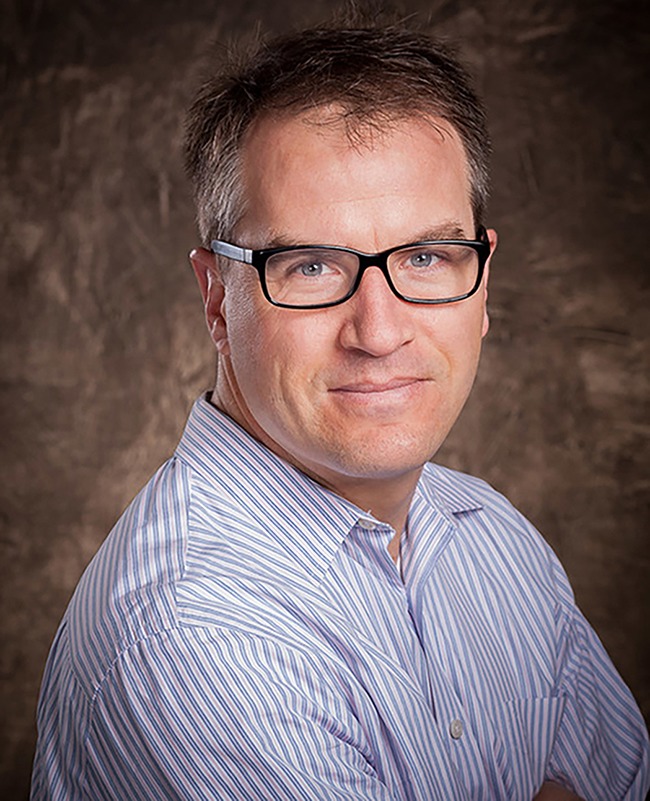


**James J. Dowling**

## Introduction

Neuromuscular diseases (NMDs) are a broad and heterogeneous collection of disorders that involve dysfunctionality of the peripheral nerves and/or muscles. For the majority of these disorders, the genetic defect has been known for decades and a vast amount of knowledge on their aetiology, epidemiology and pathophysiology is available (Emery, 2002; Mercuri and Muntoni, 2013). Although these disorders were considered untreatable for a long time, several therapeutic approaches have advanced to clinical trials in the past few years, and some have proven effective (reviewed in Dowling et al., 2017). Unfortunately, the number of NMDs for which treatment is either commercially available or available off label is very limited. The lack of treatment options is mainly due to the rarity and heterogeneity of the disorders, their often-complicated genetics, the high abundance of muscle tissue to be targeted and treated, and low treatment efficacy. With limited numbers of patients available for clinical trials due to disease rarity, compound prioritization and success rates of clinical trials likely depend on the quality and reliability of preclinical studies (Kornegay et al., 2014).

The predictive value of preclinical studies is determined by the availability of cell and animal models that can accurately recapitulate disease aspects. Initial compound selection requires cell models with corresponding genetic defects. Currently, tools such as induced pluripotent stem cells (iPSCs) and gene-editing technologies such as clustered regularly interspaced short palindromic repeats (CRISPR)/Cas9 have respectively enabled the rapid production of suitable cell and animal models (Gurumurthy and Lloyd, 2019). The generation of humanized animal models, which recapitulate aspects of the disease pathology and progression and carry the human-specific causative genetic lesion, has become easier in the past decade. Increased attention to the need for insights in natural disease history, standardization of functional outcome measures, validation of results and discovery of biomarkers has moved the field forward. Committees like the TREAT-NMD Advisory Committee for Therapeutics (TACT) have been put in place to critically evaluate preclinical data before a potential drug is further tested in clinical settings (Heslop et al., 2015; Wagner et al., 2020). This Special Issue of Disease Models & Mechanisms (DMM) focuses on all these important aspects of translational research.

## Conversations

To many researchers in the NMD field, science is personal. Therefore, we open this issue with exclusive ‘A Model for Life’ interviews with Elizabeth McNally and Louis Kunkel, two pioneers in the field who have dedicated their careers to improving our understanding of NMD biology and translating it into clinically viable interventions. Elizabeth talks about the important role of targetable genetic modifiers and about her passion for team sports (McNally, 2019), whereas Louis discusses the excitement of mapping the dystrophin gene and his family's genetics approach to gardening (Kunkel, 2019).

We also present a series of ‘First Person’ interviews with the early-career researchers who were the first authors of research articles in this special issue. In these, a new generation of scientists tells us the stories behind their papers, the key challenges of NMD research and their future career plans.

## Special articles: how to not get lost in translation

This issue features two ‘Special Articles’ that, from vastly different perspectives, highlight the steps necessary to successfully translate fundamental insights into viable clinical approaches. The first, by Belinda Cowling and Leen Thielemans (Cowling and Thielemans, 2019), provides a roadmap and valuable personal experience for researchers planning on transitioning their careers to the industry.

Next, guest editors Annemieke Aartsma-Rus and Maaike van Putten discuss the unique advantages and challenges of developing humanized mouse models for neuromuscular disease. In a field in which defined genetic causes are the backbone and foundation of disease understanding and pathogenesis, and mutation-specific forms of therapies hold great therapeutic potential, mouse models that carry the human sequence and recapitulate the key features of human disease represent a viable testing platform (Aartsma-Rus and van Putten, 2019).

## Reviews: state of the art

A new ‘At a Glance’ article presents the cellular mechanisms that govern the growth and regeneration of skeletal muscle, and highlights the defects in satellite cell function that give rise to muscular dystrophies (Morgan and Partridge, 2020). The accompanying poster, which is available in high resolution (http://dmm.biologists.org/content/13/2/dmm042192/F1.poster.jpg), visualizes the processes discussed in the article.

Improved understanding of the biological processes governing muscle development and regeneration can point to both therapeutic windows and valuable biomarkers for tracking disease progression and response to treatment. Although researchers can use various model systems to address these questions, the mouse remains the most popular model animal. van Putten and colleagues have collated a comprehensive table summarizing mouse models of various muscular dystrophies (van Putten et al., 2020), which will undoubtedly help researchers select the most appropriate mouse strain for their study. Providing an excellent example of how choosing the appropriate model system can accelerate discovery, a Review by Miranda Grounds' group discusses the biomarkers that can track myonecrosis, oxidative stress and inflammation in experimental systems of Duchenne muscular dystrophy (DMD) and in patients (Grounds et al., 2020).

Concluding this section, members of the TREAT-NMD Advisory Committee for Therapeutics review their own experiences in providing detailed feedback on clinical proposals for neuromuscular diseases submitted by researchers in both academia and industry (Willman et al., 2020). Because most individual neuromuscular disorders are rare, the patient pools are limited. This, combined with the complex aetiology and heterogeneous genetics of these disorders, emphasizes that a timely critical review of preclinical work can significantly help to de-risk translation and improve prospects for patients. The article also offers recommendations for planning preclinical studies based on ‘lessons learned’ from past experiences.

## New research: of cells, mice and much more

This Special Issue contains a large collection of research papers that utilized cell and/or animal models to study pathological features of NMDs. Two papers described novel patient-derived cell models for X-linked disorders. Perez-Siles et al. generated an iPSC line from an X-linked distal hereditary motor neuropathy (dHMNX) patient carrying a mutation in the copper transporter ATP7A (Perez-Siles et al., 2020). The derived motor neurons had reduced ATP7A levels leading to alterations in mitochondrial features. This model provides the field with a tool to further study the pathological process leading to axonal degeneration in dHMNX. Fernandes et al. derived immortalized myoblasts from an X-linked myopathy patient with a small indel mutation in the *VMA21* gene (Fernandes et al., 2020). They studied how autophagy is regulated during myogenesis in this disorder and observed uncontrolled myoblast fusion, which might explain why muscles are the predominantly involved tissue in this disorder. Patient-derived cell models provide ample opportunities to study very rare disorders in a cost-effective manner and allow a first screening for therapeutic strategies.

The availability of animal models that (at least partly) recapitulate the genetic and pathological hallmarks found in NMD patients is of great importance for the execution of more in-depth preclinical research. They not only allow for pharmacokinetic, pharmacodynamic and safety studies, but also enable assessments of a therapeutic compound's effects on muscle quality and functionality. This Special Issue contains two articles describing novel mouse models. Demonbreun et al. utilized CRISPR/Cas9 gene editing to generate a mouse model for limb-girdle muscular dystrophy type 2C (Demonbreun et al., 2019). This 521ΔT mouse carries the most frequent reading-frame-disrupting mutation found in patients: a single-nucleotide deletion in exon 6 of the *SGCG* gene. Mice consequently suffer from a severe muscular dystrophy. The authors show, for the first time, that the disrupted reading frame of the *Sgcg* gene could be restored with antisense oligonucleotide exon skipping, leading to production of internally truncated but functional γ-sarcoglycan protein upon local administration. Cordero-Sanchez et al*.* generated a knock-in mouse model (KI-STIM^I115F^) that carries a clinically relevant mutation in one of the two calcium-sensing EF-hand motifs of STIM1 (Cordero-Sanchez et al., 2019). Gain-of-function mutations in STIM1 underlie very rare disorders characterized by loss of muscle tissue and platelet dysfunctionality. The authors showed that the heterozygous mice described in the paper suffer from muscle pathology and thrombocytopenia, and as such can be used to further study these pathological features.

To allow successful preclinical development of therapeutic strategies, it is important to have access to extensive data on the natural disease progression of animal models, a broader understanding of the disease pathology, and robust outcome measures that can be used to assess treatment efficacy. Zdenka Ellederová’s group neatly characterized pathology of the brain and nerves of a minipig model for Huntington's disease (Ardan et al., 2020; Baxa et al., 2020). Their in-depth studies on several disease aspects, which were conducted at several stages throughout the animal model's life, will be of great benefit for those working with this minipig and other large animal models of Huntington's disease and facilitate the design of future intervention studies.

Although the skeletal muscle system is most dramatically affected in most NMDs, some affect multiple organs. Additionally, environmental or intrinsic factors, like altered gene expression profiles, are hypothesized to influence pathology. In DMD patients, the causative mutation results in a lack of brain-specific dystrophin isoforms, which leads to behavioural and cognitive deficits. Stay et al. neatly investigated the cerebellar circuit in the *mdx* mouse model of DMD and showed that the absence of full-length dystrophin alters the firing rate and pattern of Purkinje cells (Stay et al., 2019). Figueroa-Romero et al*.* reported that young SOD1^G93A^ mice, a model for amyotrophic lateral sclerosis (ALS), experience alterations in their microbiome and expansion and activation of immune cells prior to developing motor function deficits (Figueroa-Romero et al., 2019). O'Brien et al. showed that diet and metabolism can affect motor neuron health: in high-fat diet fed mice, an abnormal nerve-lipid signalling underlies the peripheral neuropathy seen in prediabetic and type 2 diabetic patients (O'Brien et al., 2020). Further investigations of these early alterations could help identify biomarkers and/or therapeutic interventions, and highlight the need for interdisciplinary approaches when uncovering the biology and therapeutic windows of NMDs.

The guest editors of this Special Issue have selected two Editors’ choice articles. The first pick is from Chagovetz et al. The authors elegantly dissected the function of different ryanodine receptors (RyRs) during zebrafish development, and thereby increased our understanding of the interplay between different RyR isoforms and of the pathological mechanisms underlying the heterogeneous set of phenotypes in patients with RYR1 mutations (Chagovetz et al., 2019). The second Editors’ choice article studied growth and skeletal development in several mouse models of DMD. The Editors have highlighted this article because the authors report that the *mdx:*Cmah^−/−^ mouse model for DMD is not suitable to study certain aspects of DMD pathology (Wood et al., 2020). Unfortunately, a tendency exists amongst researchers to prioritize publishing data that confirm suitability of a particular model system or reveal a beneficial effect of a drug. However, understanding whether model systems cannot be used to address a particular research question, or whether a drug target is not as promising as hypothesized, is just as crucial. As such, these negative findings are very important to move the field forward and we applaud the authors for doing so.

## From DMM’s archive

Aside from the excellent research published in this Special Issue, DMM has long had the privilege of featuring a number of excellent research and review-type articles in the field of neuromuscular disorders. Here, we highlight the most-read ones, and apologize to the authors of the articles we couldn't feature due to space restraints.

A standout article from 2018 described a novel CRISPR/Cas9-generated rabbit model of DMD by Renzi Han's group (Sui et al., 2018). Dominic Wells wrote an editorial highlighting how this new model complements existing animal models of this devastating neuromuscular disorder (Wells, 2018). CRISPR/Cas9 genome editing was also the tool of choice for Egorova et al., who used it to generate a mouse model of a newly identified *DMD* mutation in a Russian patient (Egorova et al., 2019).

As discussed above, iPSC-derived models are increasingly valuable resources in rare disease research, as also shown in the 2019 article by the Suzuki group, who investigated the role of the *C9ORF72* expansion in skeletal myocytes differentiated from ALS patient iPSCs (Lynch et al., 2019).

We also published a number of comprehensive reviews. Examples include articles on the various mouse models of ALS (De Giorgio et al., 2019), on the roles of dystroglycans (Nickolls and Bönnemann, 2018) and collagen VI (Gregorio et al., 2018) in the nervous and muscular systems, and on the most recent insights from core myopathy model systems (Fusto et al., 2019).

This Special Issue also launches an ongoing subject collection (https://dmm.biologists.org/collection/neuromuscular), in which we will continue to collate exciting review, research and resource articles. We hope you enjoy reading these freely accessible articles.

## Conclusions

The Special Issue nicely highlights the amazing advances in the neuromuscular field in recent years related to pathomechanistic understanding and therapy development. There is an increasing amount of novel cell and animal models available, even for some of the rarest NMDs, which will allow for further characterization of the disease pathology and hopefully facilitate the development of novel therapeutic strategies. It has become clear that insights into the natural history of the individual NMDs, standardization of outcome measures and validation of research findings are of utmost importance to de-risk translation of therapeutic efficacy from model systems to patients. This change in perspective could move the NMD field forward and might result in better selection of candidate compounds and eventually a higher success rate in clinical trials for these devastating disorders.

This article is part of a special collection ‘A Guide to Using Neuromuscular Disease Models for Basic and Preclinical Studies’, which was launched in a dedicated issue guest edited by Annemieke Aartsma-Rus, Maaike van Putten and James Dowling. See related articles in this collection at http://dmm.biologists.org/collection/neuromuscular.
